# Continental data on cave-dwelling spider communities across Europe (Arachnida: Araneae)

**DOI:** 10.3897/BDJ.7.e38492

**Published:** 2019-10-08

**Authors:** Stefano Mammola, Pedro Cardoso, Dorottya Angyal, Gergely Balázs, Theo Blick, Hervé Brustel, Julian Carter, Srećko Ćurčić, Samuel Danflous, László Dányi, Sylvain Déjean, Christo Deltshev, Mert Elverici, Jon Fernández, Fulvio Gasparo, Marjan Komnenov, Christian Komposch, L’ubomír Kováč, Kadir Boğaç Kunt, Andrej Mock, Oana Moldovan, Maria Naumova, Martina Pavlek, Carlos E. Prieto, Carles Ribera, Robert Rozwałka, Vlastimil Růžička, Robert S. Vargovitsh, Stefan Zaenker, Marco Isaia

**Affiliations:** 1 Department of Life Sciences and Systems Biology, University of Turin, Turin, Italy Department of Life Sciences and Systems Biology, University of Turin Turin Italy; 2 Laboratory for Integrative Biodiversity Research (LIBRe), Finnish Museum of Natural History, University of Helsinki, Helsinki, Finland Laboratory for Integrative Biodiversity Research (LIBRe), Finnish Museum of Natural History, University of Helsinki Helsinki Finland; 3 Department of Zoology, Hungarian Natural History Museum, Budapest, Hungary Department of Zoology, Hungarian Natural History Museum Budapest Hungary; 4 UMDI, Faculty of Sciences, UNAM National Autonomous University of Mexico, Sisal, Mexico UMDI, Faculty of Sciences, UNAM National Autonomous University of Mexico Sisal Mexico; 5 Department of Systematic Zoology and Ecology, Eötvös Loránd University, Budapest, Hungary Department of Systematic Zoology and Ecology, Eötvös Loránd University Budapest Hungary; 6 Independent Researcher, Hummeltal, Germany Independent Researcher Hummeltal Germany; 7 Ecole d'Ingénieur de Purpan, Toulouse, France Ecole d'Ingénieur de Purpan Toulouse France; 8 Amgueddfa Cymru National Museum Wales, Cardiff, United Kingdom Amgueddfa Cymru National Museum Wales Cardiff United Kingdom; 9 Faculty of Biology, Institute of Zoology, University of Belgrade, Belgrade, Serbia Faculty of Biology, Institute of Zoology, University of Belgrade Belgrade Serbia; 10 Conservatoire d'Espaces Naturels de Midi-Pyrénées, Toulouse, France Conservatoire d'Espaces Naturels de Midi-Pyrénées Toulouse France; 11 Conservatoire d'Espaces Naturels de Midi-Pyrénées, Tolouse, France Conservatoire d'Espaces Naturels de Midi-Pyrénées Tolouse France; 12 National Museum of Natural History, Bulgarian Academy of Sciences, Sofia, Bulgaria National Museum of Natural History, Bulgarian Academy of Sciences Sofia Bulgaria; 13 Department of Biology, Faculty of Science and Arts, Erzincan Binali Yıldırım University, Erzincan, Turkey Department of Biology, Faculty of Science and Arts, Erzincan Binali Yıldırım University Erzincan Turkey; 14 Independent researcher, Basque Country, Spain Independent researcher Basque Country Spain; 15 Commissione Grotte “E. Boegan”, Società Alpina delle Giulie, C.A.I., Trieste, Italy Commissione Grotte “E. Boegan”, Società Alpina delle Giulie, C.A.I. Trieste Italy; 16 Independent Researcher, Blwd Kuzman Josifovski Pitu, Skopje, Macedonia Independent Researcher, Blwd Kuzman Josifovski Pitu Skopje Macedonia; 17 OEKOTEAM - Institute for Animal Ecology and Landscape Planning, Graz, Austria OEKOTEAM - Institute for Animal Ecology and Landscape Planning Graz Austria; 18 Institute of Biology and Ecology, Faculty of Science, Pavol Jozef Šafárik University, Košice, Slovakia Institute of Biology and Ecology, Faculty of Science, Pavol Jozef Šafárik University Košice Slovakia; 19 Zoological Collection of Cyprus Wildlife Research Institute, Kyrenia, Cyprus Zoological Collection of Cyprus Wildlife Research Institute Kyrenia Cyprus; 20 Department of Biology, Faculty of Science, Eskişehir Technical University, Eskişehir, Turkey Department of Biology, Faculty of Science, Eskişehir Technical University Eskişehir Turkey; 21 Emil Racovitza Institute of Speleology, Cluj-Napoca, Romania Emil Racovitza Institute of Speleology Cluj-Napoca Romania; 22 Institute of Biodiversity and Ecosystem Research, Bulgarian Academy of Sciences, Sofia, Bulgaria Institute of Biodiversity and Ecosystem Research, Bulgarian Academy of Sciences Sofia Bulgaria; 23 Ruđer Bošković Institute, Zagreb, Croatia Ruđer Bošković Institute Zagreb Croatia; 24 Croatian Biospeleological Society, Zagreb, Croatia Croatian Biospeleological Society Zagreb Croatia; 25 Department of Evolutionary Biology, Ecology and Environmental Sciences & Biodiversity Research Institute, University of Barcelona, Barcelona, Spain Department of Evolutionary Biology, Ecology and Environmental Sciences & Biodiversity Research Institute, University of Barcelona Barcelona Spain; 26 Department of Zoology & Animal Cell Biology, Faculty of Science and Technology, University of the Basque Country, Bilbao, Spain Department of Zoology & Animal Cell Biology, Faculty of Science and Technology, University of the Basque Country Bilbao Spain; 27 Faculty of Biology and Environmental Sciences, Cardinal Stefan Wyszyński University, Warsaw, Poland Faculty of Biology and Environmental Sciences, Cardinal Stefan Wyszyński University Warsaw Poland; 28 Biology Centre, Institute of Entomology, České Budějovice, Czech Republic Biology Centre, Institute of Entomology České Budějovice Czech Republic; 29 Schmalhausen Institute of Zoology, National Academy of Sciences of Ukraine, Kiev, Ukraine Schmalhausen Institute of Zoology, National Academy of Sciences of Ukraine Kiev Ukraine; 30 Verband der deutschen Höhlen- und Karstforscher e.V., Fulda, Germany Verband der deutschen Höhlen- und Karstforscher e.V. Fulda Germany

**Keywords:** Araneae, cave, Europe, spiders, subterranean biology, troglophile, troglobiont

## Abstract

**Background:**

Spiders (Arachnida: Araneae) are widespread in subterranean ecosystems worldwide and represent an important component of subterranean trophic webs. Yet, global-scale diversity patterns of subterranean spiders are still mostly unknown. In the frame of the CAWEB project, a European joint network of cave arachnologists, we collected data on cave-dwelling spider communities across Europe in order to explore their continental diversity patterns. Two main datasets were compiled: one listing all subterranean spider species recorded in numerous subterranean localities across Europe and another with high resolution data about the subterranean habitat in which they were collected. From these two datasets, we further generated a third dataset with individual geo-referenced occurrence records for all these species.

**New information:**

Data from 475 geo-referenced subterranean localities (caves, mines and other artificial subterranean sites, interstitial habitats) are herein made available. For each subterranean locality, information about the composition of the spider community is provided, along with local geomorphological and habitat features. Altogether, these communities account for > 300 unique taxonomic entities and 2,091 unique geo-referenced occurrence records, that are made available via the Global Biodiversity Information Facility (GBIF) (Mammola and Cardoso 2019). This dataset is unique in that it covers both a large geographic extent (from 35° south to 67° north) and contains high-resolution local data on geomorphological and habitat features. Given that this kind of high-resolution data are rarely associated with broad-scale datasets used in macroecology, this dataset has high potential for helping researchers in tackling a range of biogeographical and macroecological questions, not necessarily uniquely related to arachnology or subterranean biology.

## Introduction

Spiders (Arachnida: Araneae) are widespread in caves and other subterranean ecosystems worldwide, representing an important component of subterranean trophic webs (Deharveng and Bedos 2019). They are distinctive for their key ecological role as predators and for the variety of functional adaptations, representing therefore ideal model organisms for exploring a variety of ecological and evolutionary topics (Mammola and Isaia 2017). For example, different spider species have been used for studying silk's mechanical and structural properties (Lepore et al. 2012, Piorkowski et al. 2017), for exploring a range of morphological, metabolic and behavioural adaptations (Cardoso and Scharff 2009, Doran et al. 2001, Doran et al. 2017, Hadley et al. 1981, Lipovšek et al. 2018, Lipovšek et al. 2017, Miller 2005, Yancey et al. 2018, Chiavazzo et al. 2015, Michalik et al. 2014,Hesselberg et al. 2019), for shedding light on the mechanisms of speciation and the processes underpinning biological radiations (Arnedo et al. 2007, Hedin 2015, Růžička et al. 2013, Yao et al. 2016, Zhang and Li 2013), as well as for testing ecological hypotheses (Cardoso 2012, Mammola et al. 2016, Mammola et al. 2019, Novak et al. 2010, Lunghi 2018).

Yet, the accessible information about the ecology of most subterranean spiders is still limited, especially when considering broad-scale spatial and temporal patterns of subterranean communities (that is, a macroecological perspective). Indeed, due to the general paucity of information on most subterranean spiders (e.g. Huber 2018, Mammola et al. 2018, Cardoso 2012) and the lack of broad-scale databases about their distribution (Culver et al. 2013, Mammola 2019), global-scale diversity patterns of subterranean spiders remain virtually undescribed (Mammola et al. 2018a, Mammola and Isaia 2017). In an attempt to overcome this impediment, we created an international network of araneologists and cavers (that we called the "CAWEB" network; Mammola et al. 2017) to compile the first continental-scale geo-referenced dataset of cave-dwelling and other subterranean spider communities (Mammola et al. 2019b). In this data paper, we describe these datasets and make them freely available online for future use. We aim to provide an accessible tool for exploring continental patterns of subterranean species distribution, as well as to further expand the CAWEB network and thus the geographical coverage of these datasets.

## Geographic coverage

### Description

Europe.

### Coordinates

35.0 and 67.0 Latitude; –9.0 and 37.0 Longitude.

## Taxonomic coverage

### Taxa included

**Table:** 

Rank	Scientific Name	Common Name
order	Araneae	Spiders

## Usage rights

### Use license

Creative Commons Public Domain Waiver (CC-Zero)

## Data resources

### Data package title

Cave_dwelling_spiders_Europe

### Number of data sets

3

### Data set 1.

#### Data set name

GBIF_occurrence_cave_spiders

#### Data format

Tab delimited file (.csv).

#### Number of columns

22

#### Download URL

https://doi.org/10.15468/ygocko

#### Description

A dataset with all the referenced distribution points of the species considered in the subterranean localities included in the CAWEB project. This biodiversity dataset is constructed following the Darwin Core standard.

**Data set 1.** 

Column label	Column description
id	An alphanumeric identifier ("Ara" followed by a progressive number; e.g. Ara0001) for the Occurrence (as opposed to a particular digital record of the occurrence).
basisOfRecord	The specific nature of the data record. Categorical vairable. Either 'PreservedSpecimens' (data record based on specimens stored in a museum or private collection), 'Literature' (data record based on literature information) or HumanObservation (data record based on personal observations by the author of each records). See "notes" and "referencesSpecies" columns in the "Cave description.csv" dataset for full bibliographic details.
collectionCode	For "PreservedSpecimens", the name identifying the collection or dataset from which the record was derived. Note that, in spelling institutions and collection names, we have omitted accents (e.g. á, è, ò) and special characters (e.g. ä, č, ê) in order to avoid formatting problems.
informationWithheld	Additional information relative to each record, indicating the person to contact for information about the record. Note that, in spelling contact names, we have omitted accents (e.g. á, è, ò) and special characters (e.g. ä, č, ê) in order to avoid formatting problems.
datasetName	The name of the dataset from which the record was derived.
bibliographicCitation	The bibliographic reference for the resource, indicating how individual records should be cited (attributed) when used.
country	The name of the country or major administrative unit in which the verbatimLocality is situated.
locationID	An identifier for the set of location information. Same as the column ID in the "Cave_description" dataset.
verbatimLocality	The original textual description of the locality.
decimalLatitude	The geographic latitude (in decimal degrees, using the spatial reference system given in geodeticDatum) of the geographic centre of a location.
decimalLongitude	The geographic longitude (in decimal degrees, using the spatial reference system given in geodeticDatum) of the geographic centre of a location.
geodeticDatum	The ellipsoid, geodetic datum or spatial reference system (SRS) upon which the geographic coordinates, given in decimalLatitude and decimalLongitude, are based.
georeferenceProtocol	A description or reference to the methods used to determine the spatial footprint, coordinates and uncertainties.
phylum	The full scientific name of the phylum or division in which the taxon is classified.
class	The full scientific name of the class in which the taxon is classified.
order	The full scientific name of the order in which the taxon is classified.
family	The full scientific name of the family in which the taxon is classified.
genus	The full scientific name of the genus in which the taxon is classified.
specificEpithet	Specific epithet of the taxonomic record.
specificName	The full scientific name, with authorship and date information if known.
scientificNameAuthorship	The authorship information for the scientific name formatted according to the conventions of the applicable nomenclatural code.
taxonRank	The highest taxonomic rank in the specificName – either a genus or a species.

### Data set 2.

#### Data set name

Cave_description

#### Data format

Tab delimited file (.csv)

#### Number of columns

25

#### Download URL

10.6084/m9.figshare.8224025

#### Description

A dataset with all the information about the subterranean localities included in the CAWEB project. The R notation 'NA' is used for missing values.

**Data set 2.** 

Column label	Column description
ID	An alphanumeric identifier ("CAVE_" followed by a progressive number; e.g. CAVE_001) for the subterranean locality. Note that the exact same "ID" is used in the "Community_composition" dataset, in order to unambiguously link each subterranean locality with its spider community's composition.
locality	Name of the cave/subterranean locality. Not translated in English.
country	The name of the country or major administrative unit in which the subterranean locality is situated.
decimalLongitude	The geographic longitude of the entrance of the subterranean locality.
decimalLatitude	The geographic latitude of the entrance of the subterranean locality.
geodeticDatum	The ellipsoid, geodetic datum or spatial reference system (SRS) upon which the geographic coordinates given in decimalLatitude and decimalLongitude are based.
elevation	Altitude a.s.l. of the subterranean locality's main entrance in metres (m).
aspect	The direction that the main entrance of the cave/subterranean locality faces. Categorical variables. N = North; S = South; E = East; W = West; flat = entrance in a plane terrain.
entranceNumber	Number of known subterranean localities' entrances (if any).
entranceType	The general morphology of the subterranean locality's main entrance. Categorical variables. ascendent = ascending; descendent = descending entrance; horizontal = horizontal entrance; pit = vertical entrance.
entranceSize	Size (base x height) of the subterranean locality's main entrance in square metres (m^2^).
entranceHabitat	Prevalent habitat in which the subterranean locality opens. Categorical variables. Either "agricultural", "forest", "grass", "rocky", "shrubs" or "urbanized".
entranceHabitatVerbatim	A verbatim description of the habitat in which the subterranean locality opens.
development	The subterranean locality total planimetric development in metres (m).
positiveDrop	Total ascent of the subterranean locality in metres (m).
negativeDrop	Total descent of the subterranean locality in metres (m).
caveType	The type of subterranean locality. Categorical variable. Either "artificial" (e.g. mine, mineshafts, military bunkers, railways, subterranean blockhouses, cellars etc.), "ialine" (ialine caves), "ice" (ice caves), "karst" (karst caves, dolines etc.), "other" (other types; e.g. interstitial habitats), "tectonic" (talus caves, cracks, faults etc.), "volcanic" (volcanic caves, lava tubes etc.).
caveMorphology	The general morphology of the subterranean locality (i.e. prevalent morphology along the locality). Categorical variables. ascendent = prevalently an ascending morphology; descendent = prevalently a descending morphology; horizontal = prevalently a horizontal morphology; pit = primarily a vertical pit/abyss.
caveActive	Binary variable. If the subterranean locality is active (1) or not (0). An active cave is a cave which has a stream flowing in it.
caveTouristic	Binary variable. If the subterranean locality is open to general tourists (1) or not (0).
notes	Additional notes about the subterranean locality.
referencesLocality	References with additional information about the subterranean locality (if any).
referencesSpecies	References with additional information about the spider species reported for the subterranean locality (if any).
contributorName	Name(s) of the person(s) who contributed information about the subterranean locality. Note that, in spelling contributors names, we have omitted accents (e.g. á, è, ò) and special characters (e.g. ä, č, ê) in order to avoid formatting problems.
contributorEmail	E-mail adress(es) of the person(s) who contributed information about the subterranean locality.

### Data set 3.

#### Data set name

Community_composition

#### Data format

Tab delimited file (.csv).

#### Number of columns

9

#### Download URL

10.6084/m9.figshare.8224025

#### Description

A dataset with the spider community composition (species presence/absence data) of each subterranean locality included in the CAWEB project.

**Data set 3.** 

Column label	Column description
Family	The full scientific name of the family in which the taxon is classified.
Genus	The full scientific name of the genus in which the taxon is classified.
Species	Species epithet of the scientificName.
Author	The authorship information for the scientificName formatted according to the conventions of the applicable nomenclaturalCode.
specificName	Genus and species combined together.
taxonRank	The highest taxonomic rank available (either genus or species).
Adaptation	Habitat preference of the species. Note that accidental species are not included in the dataset – full details in Mammola et al. (2018a). Binary variable. Either troglobiont (1) or troglophile (0).
species_lsid	Unique Life Science Identifier (LSID) for the taxon, based on the World Spider Catalog (doi: 10.24436/2). The LSID allows a user to keep track of taxonomical changes in the status of species or link together datasets regardless of taxonomical changes.
Alphanumeric codes (CAVE_number) in progressive order	Each column after the first eight columns is labelled with an alphanumeric identifier ("CAVE_", followed by a progressive number; e.g. CAVE_001), referring to the subterranean locality as in the column "ID" of the "Community_composition" dataset. For each Genus_species in the dataset, the presence (1) or absence (0) within the subterranean locality is indicated.

## Additional information

**Figure 1.**
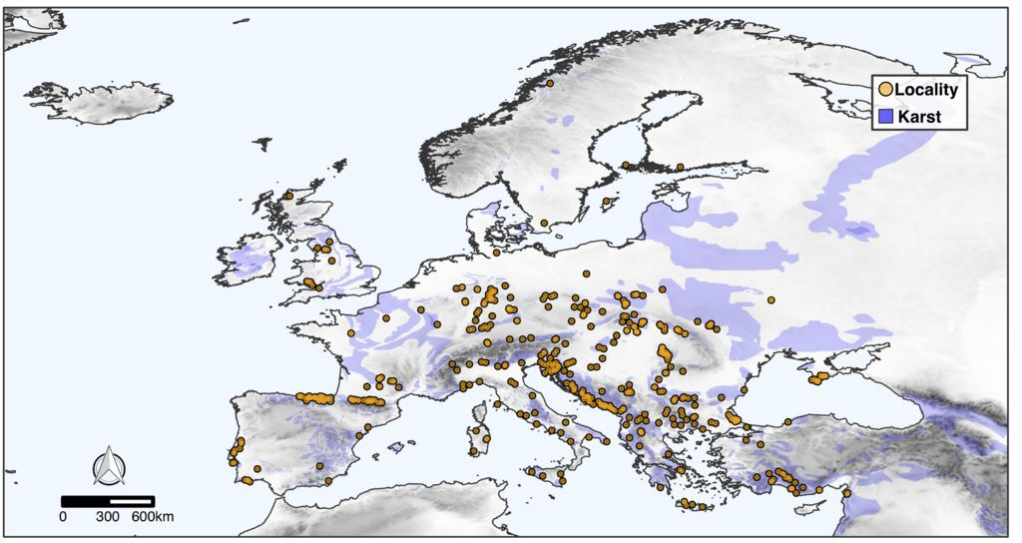
Distribution of the subterranean localities in Europe included in the CAWEB dataset. Shades of grey represent altitude. Light blue patches are karst rocks, based on the World Map of Carbonate Rock Outcrops (version 3.0).

**Figure 2.**
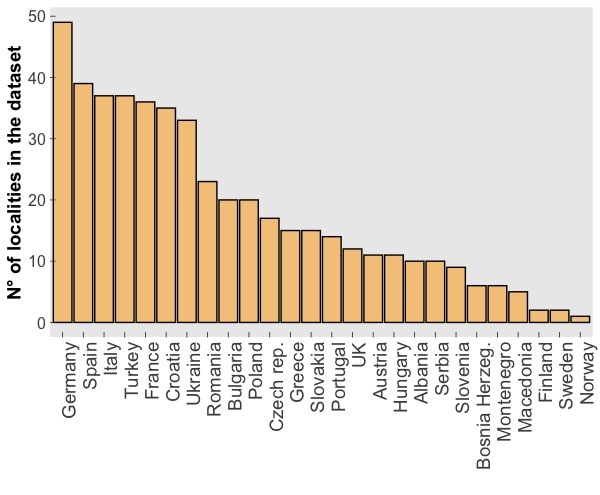
Number of subterranean localities included in the CAWEB dataset for each European country.

**Figure 3.**
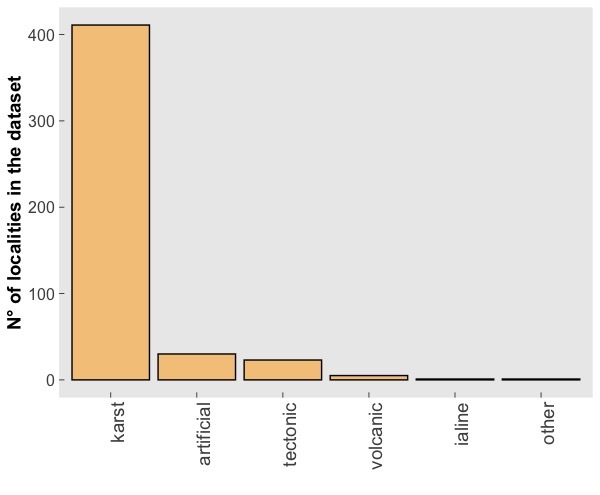
Number of records included in the dataset for each typology of subterranean locality.

The CAWEB dataset comprises data for 475 subterranean localities (Fig. 1) in 27 European countries (Fig. 2). Spider communities refer to different types of caves (karst, talus, volcanic and ialine caves), artifical subterranean sites (mines, blockhouses, cellars etc.), as well as interstitial habitats. However, it is worth noting that the majority of records are from karst caves (Fig. 3), a typical bias in subterranean datasets (Mammola and Leroy 2018, Zagmajster et al. 2010, Niemiller and Zigler 2013, Christman and Culver 2002). These localities open in different types of habitats, with a prevalence of forests and shrublands (Fig. 5).

**Figure 4.**
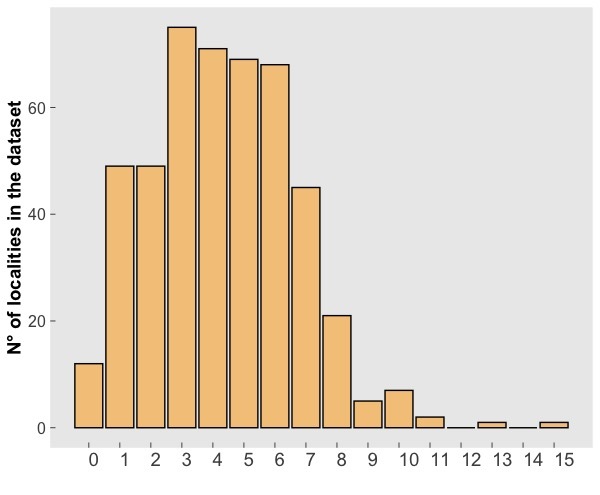
Spider species richness in subterranean localities of the CAWEB dataset. Species richness is expressed as the number of species + morphospecies.

**Figure 5.**
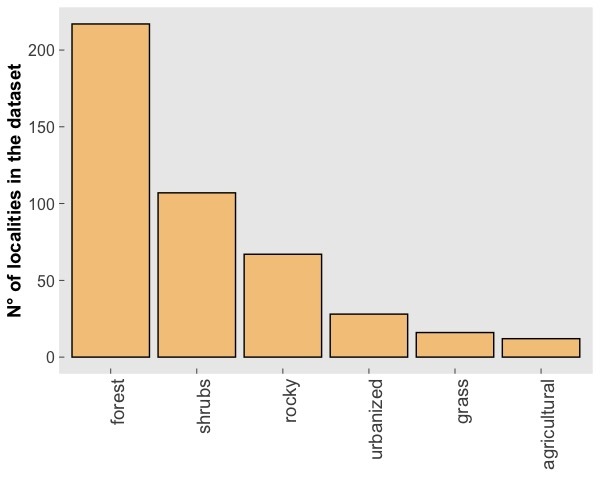
Number of records included in the CAWEB dataset for each typology of habitat at the entrance.

Subterranean localities included in the dataset account for over 300 spider species, that is more than half of the subterranean spider diversity in Europe (Mammola et al. 2018a). The number of spider species per cave ranges from 0 to 15 (mean= 4.3, s.d.= 2.35; Fig. 4). Altogether, these species account for 2,091 unique geo-referenced occurrence records across Europe. While most of the species in the dataset are recorded from one or a few caves, some troglophile species are more widely represented in the dataset (Fig. 6).

The over-arching goal of the CAWEB project was to assemble a continental dataset with information about the spider community composition of subterranean localities across the European latitudinal range. This dataset also contains local data on geomorphological and habitat features of these localities. Similar high-resolution data are rarely associated with broad-scale datasets used for macroecological analyses. Therefore, the CAWEB dataset can be used to explore a range of biogeographical and macroecological questions, potentially extending beyond arachnology and subterranean biology (see Mammola et al. 2019b for an example).

## Figures and Tables

**Figure 6a.**
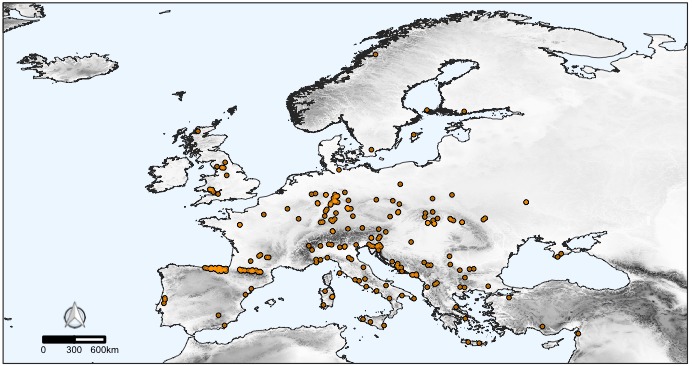
Distribution of the troglophile spider *Metellina merianae* (Scopoli, 1763) (Araneae: Tetragnathidae) in Europe, based on 238 occurrences. *Metellina merianae* is a common inhabitant of the twilight zone of caves, although it possesses rather poor adaptations to the subterrranean conditions (Hesselberg and Simonsen 2019).

**Figure 6b.**
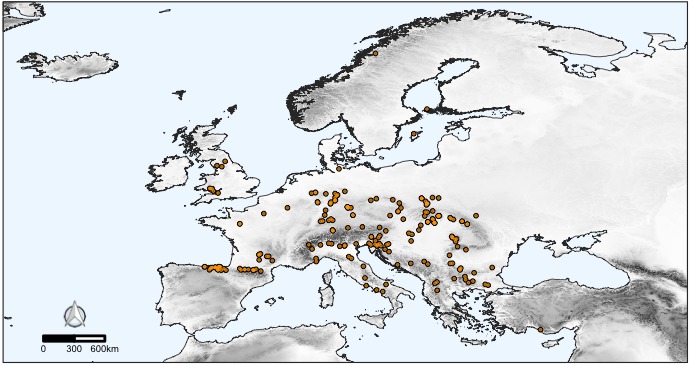
Distribution of the troglophile spider *Meta menardi* (Latreille, 1804) (Araneae: Tetragnathidae) in Europe, based on 211 occurrences. *Meta menardi* is probably the most well-known species in the twilight zone of European caves (Hesselberg et al. 2019, Mammola and Isaia 2014), where it often coexists with *Metellina merianae* (Novak et al. 2010).

**Figure 6c.**
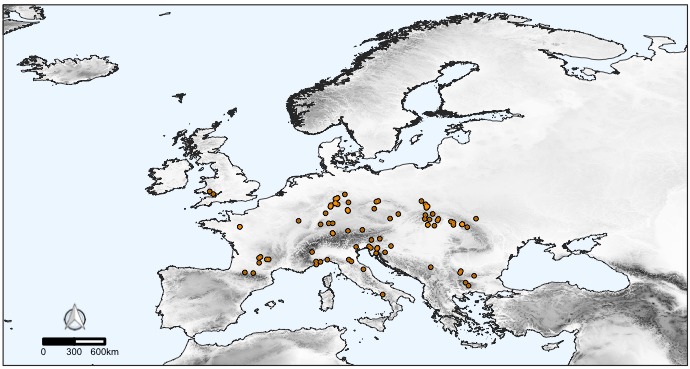
Distribution of the troglophile spider *Tegenaria silvestris* L. Koch, 1872 (Araneae: Agelenidae) in Europe, based on 92 occurrences. This species is a frequent inhabitant of shallow cave sectors (Mammola et al. 2018a).

**Figure 6d.**
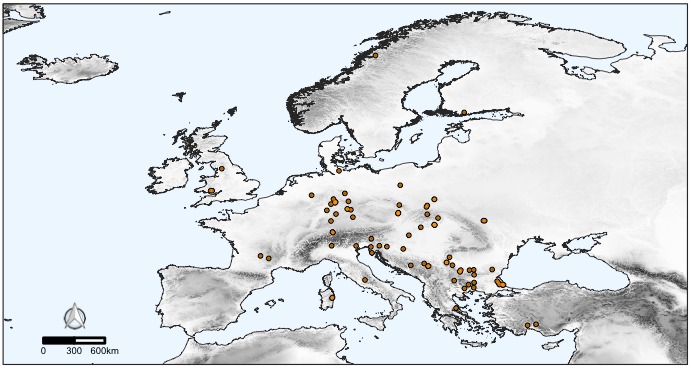
Distribution of the troglophile spider *Porrhomma convexum* (Westring, 1851) (Araneae: Linyphiidae), based on 86 occurrences. This species inhabits caves, mines and other mesic habitats up to the alpine level. It is widely distributed in Europe (Nentwig et al. 2019).
